# Ecosystem Services from Edible Insects in Agricultural Systems: A Review

**DOI:** 10.3390/insects8010024

**Published:** 2017-02-17

**Authors:** Charlotte L. R. Payne, Joost Van Itterbeeck

**Affiliations:** 1 Conservation Science Group, Department of Zoology, University of Cambridge, Cambridge CB2 3QY, UK; 2 College of Arts, Department of History, Rikkyo University, Tokyo 171-8501, Japan; joostvanitterbeeck@hotmail.com

**Keywords:** edible insects, ecosystem services, conservation, food security, entomophagy, agricultural change, soil management, pollination, pest control, agroecology

## Abstract

Many of the most nutritionally and economically important edible insects are those that are harvested from existing agricultural systems. Current strategies of agricultural intensification focus predominantly on increasing crop yields, with no or little consideration of the repercussions this may have for the additional harvest and ecology of accompanying food insects. Yet such insects provide many valuable ecosystem services, and their sustainable management could be crucial to ensuring future food security. This review considers the multiple ecosystem services provided by edible insects in existing agricultural systems worldwide. Directly and indirectly, edible insects contribute to all four categories of ecosystem services as outlined by the Millennium Ecosystem Services definition: provisioning, regulating, maintaining, and cultural services. They are also responsible for ecosystem disservices, most notably significant crop damage. We argue that it is crucial for decision-makers to evaluate the costs and benefits of the presence of food insects in agricultural systems. We recommend that a key priority for further research is the quantification of the economic and environmental contribution of services and disservices from edible insects in agricultural systems.

## 1. Introduction

There is growing concern about our ability to feed our rising population. Population trends suggest that there will be 9.6 billion people on the planet by 2050 [1], with food demand growing by 60% [2]. Thus, a concerted effort is underway to seek new ways of increasing food production [3]. Clearing more land for agriculture is considered an untenable solution, since land clearance has been linked to major biodiversity loss and climatic change [4], and latitudes with high agricultural potential are also home to high levels of biodiversity. Agricultural intensification is often portrayed as the only other option open to us, as it can facilitate increased food production while also sparing land for wildlife and carbon sequestration [5].

### 1.1. The Value of an Ecosystem Services Approach to Agricultural Systems

Yet measurement of the efficacy of agricultural intensification tends to focus on the quantity of commodity crops produced, without consideration of several other key dependent variables [6]. These often-overlooked variables include: (1) The abundance and nutrient content of additional crops essential for health such as fruit and vegetables; (2) The abundance of edible and non-edible byproducts of lower-intensity farming systems; (3) How (or whether) agricultural intensification improves the health of those suffering from nutritional deficiencies; (4) Who reaps the economic benefits of agricultural intensification. Edible insects harvested from agricultural land are a significant example of the second of these variables—an edible byproduct of lower intensity systems—and their presence or absence is likely to have an impact on all of the others. For example, edible insects tend to be high in essential micronutrients (variable 1), and are likely to be used as both a protein source (variable 3) and a source of income (variable 4) by smallholder farmers who gather them. They are often a highly valued food source in such regions, unhindered by the cultural aversion to insect foods often emphasised in a Western context [7] and enjoyed primarily for their sensory properties [8].

An ecosystem services approach is one way of identifying these and other impacts, which would not be identified through measuring plant crop yields alone. Ecosystem services have been defined as ‘the benefits ecosystems provide to people’ [9], and this is still the most common definition used today [10]. Ecosystem services are usually divided into four (or three) categories: provisioning, regulating, maintaining or supporting (sometimes subsumed into the ‘regulating’ category), and cultural services. Recognizing the value of these services is crucial for identifying the agricultural strategy that is most appropriate in any given context [11].

### 1.2. Edible Insects in Agricultural Systems

Edible insects are one of the many byproducts of low-intensity agriculture, and are usually a seasonal and protein-rich food. Their collection and processing requires low energy expenditure, and they are consumed locally and/or sold at profit. Agricultural intensification, when it comprises mechanization, tree clearance, and pesticide use, threatens the existence of many edible insects. This is particularly true for edible insects that are sold and consumed in significant quantities, and are therefore those most crucial to ensuring food security.

At least 108 countries have a tradition of consuming edible insects, and the number of native species consumed within each country ranges from only one (e.g., France) to as many as 450 (Mexico) [12]. However, even in regions where a large number of species are eaten, it is often the case that a very small proportion of these seem to account for the majority of insect consumption. Since insect consumption per person has not yet been satisfactorily quantified in any context, our most reliable indicator of consumption comes from market trends, using commercial availability as a proxy for consumption. This also indicates that these few species account for a large proportion of the economic profit made from trade in edible insects. Table 1 lists the key examples that are reported in current literature:

Due to the broad scope of the topic, we chose to conduct this review as a narrative overview of the literature, combining data from published literature with knowledge gained from our own field experience and consultation with fellow researchers in this field [26]. We used a theory-driven thematic analysis [27], and structure our review according to categories of ecosystem services, and disservices, promoted by the Millenium Ecosystem Services Assessment and reinforced in consequent literature. We chose this theory driven structure to help minimise selection bias. The resulting paper highlights important areas commonly overlooked by publications focusing on either ecosystem services or edible insects. We deemed this approach to be more appropriate than a systematic review. This is firstly because we cover a range of issues too broad for the focused approach required by a systematic review, and secondly because there is a dearth of high quality quantitative data on this topic, precluding current opportunities for meta-analysis. We hope that this review alerts others to some of the gaps and opportunities in this field.

**Figure 1:**
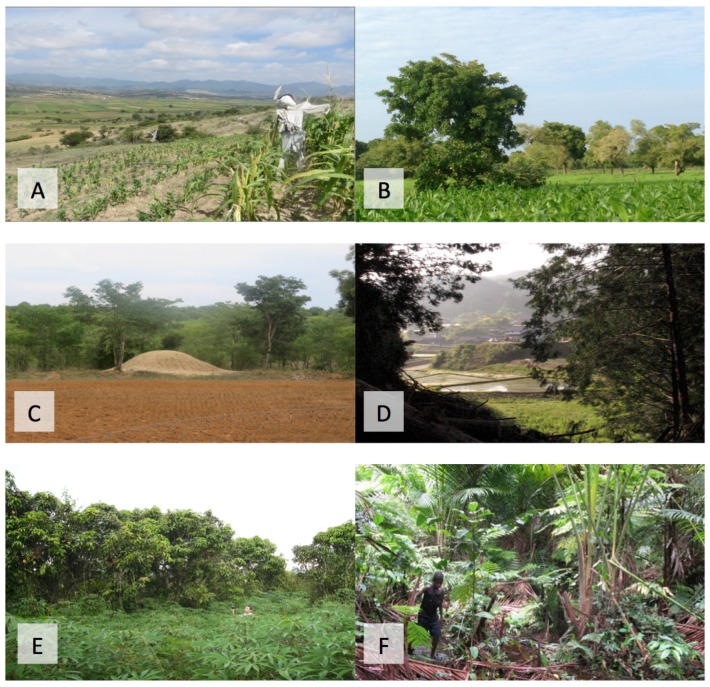
Examples of agricultural systems with edible insects: (**A**) Maize fields in Oaxaca, Mexico, where edible grasshoppers (*Sphenarium purpurascens*) are harvested; (**B**) Agroforestry (mixed maize *Zea mays* and shea *Vitellaria paradoxa*) fields in western Burkina Faso, where edible caterpillars (*Cirina butyrospermi*) are harvested; (**C**) A freshly ploughed field with a termite mound in northeastern Zimbabwe, where termites (*Macrotermes* spp.) are harvested; (**D**) A ‘satoyama’ (mixed paddyfield and forest) landscape in Japan, where edible wasps (*Vespula* spp.), hornets (*Vespa mandarinia japonica*) and grasshoppers (*Oxya* spp.) are harvested; (**E**) A mango and papaya plantation in Thailand where weaver ants (*Oecophylla smaragdina*) are harvested; (**F**) A palm plantation in Papua New Guinea where palm weevil larvae (*Rhynchophorus* spp.) are harvested. (*Photos A–D by Charlotte Payne, photo E by Joost Van Itterbeeck, photo F by Kenichi Nonaka*).

We limited the scope of this review to include only ecosystem services provided by insects within existing agricultural systems. Figure 1 illustrates some of these systems. We exclude those edible insect species of high nutritional and economic importance that are collected only from wild ecosystems, and those that are reared in enclosed systems, although these too no doubt provide multiple ecosystem services. We do include the *Bombyx mori* silkworm, which is bred primarily for silk production and its cultivation requires that large areas of land are devoted to mulberry plantations, therefore the resulting edible product is part of an existing agricultural system. Overall, the species and systems we discuss are necessarily constrained by the literature available to us; for example, we found very little published research on the role of edible aquatic insects in agricultural systems.

### 1.3. The Value of Insects in Agricultural Systems for the Economy and for Food Security

Insects are an integral part of agricultural systems worldwide, and are recognized as being of considerable economic importance. Losey and Vaughan [28], for example, estimated in 2009 that wild insects were worth $57 billion per year in the US. They define wild insects as those that are not domesticated or mass-bred in enclosed systems, therefore encompassing both those insects that are found in non-cultivated landscapes and those that range freely in existing agricultural systems.

Losey and Vaughan [28] reviewed the ecosystem services provided by wild insects and focused on dung burial (a regulating/supporting service), pest control (a regulating/supporting service), pollination (a regulating/supporting service), and wildlife nutrition (a provisioning service). All of these concern insects within agricultural systems. Here, we offer a global perspective, with a focus on insects that already provide provisioning services to human communities through their role of food. Our review is necessarily skewed by the nature of data that is available in English language peer-reviewed publications, and we have selected for inclusion in this article those examples that are best represented in the literature. We consider the role of these insects in offering provisioning, regulating, maintaining, and cultural services.

This is important because insect eating habits and land use patterns are changing, and we risk losing many of the valuable ecosystem services provided by edible insects as a result. A dietary shift towards a more westernized diet, accompanied by a population shift to urban areas, has in many countries led to a decline in the consumption of traditional foods in regions with a long history of insect consumption [29,30], although an exception to this is found in parts of Southeast Asia [31]. Meanwhile, the clearance of wild land and the adoption of agrochemical use by traditional farmers threaten the habitats in which many edible insects are found, jeopardizing both their safety as a food source and their future availability [30,32]. In 2013, an influential report by the Food and Agriculture Organization of the United Nations (FAO) highlighted the potential of insects as food [33]. The report included a section on insects as a natural resource, and mentioned the importance of ecosystem services provided by edible insects, including those in existing agricultural systems. Yet the main focus of the report, and indeed its main impact to date, has been to stimulate growth in the farming of edible insects in high density, enclosed systems [34]. With this review we hope to reopen the discussion about the importance of proactive management for conserving edible insects in existing agricultural systems, in order to ensure that future generations also benefit from the ecosystem services that they provide.

## 2. Provisioning Services

Provisioning services are those that provide goods for direct human use, and which are often part of the economy [9]. Edible insects in agricultural systems provide food and income.

### 2.1. Food

All edible insects are a potential source of food for humans. The most recent list counts 2141 different species [35], but given uncertain identification, the neglect of research into the dietary repertoire of many ethnic groups and the possible inclusion of synonyms among others, the actual number of species may differ significantly from this value. Hard data, and reliable data, on consumption levels and actual nutritional contribution are scarce. The lack of this type of data is a severe impediment to understanding the true significance of insects as a food source in peoples’ current diets.

As a food, insects are consumed in various ways. They can be served alongside regional staples as the main source of protein in a complete meal. This is commonly the case with the shea caterpillar *Cirina* spp. in West Africa (usually served in a tomato sauce with rice or maize meal, or as a sandwich filling) and the grasshopper *Sphenarium purpurascens* (‘*chapulines*’) in Mexico (usually served with maize tortillas). In Thailand, insects are mainly eaten as snacks, including deep-fried grasshoppers (various species), water beetles (various species), and bamboo caterpillars (*Omphisa fuscidentalis*) [36]. In Laos, a paste is made of crickets (*Brachytrupes portentosus*), stinkbugs (*Tessaratoma quadrata*), and giant water bugs (*Lethocerus indicus*), amongst others, with other condiments mixed in [37,38]. This is used as a dip which makes the dry glutinous rice more palatable. Also in Laos, the larvae and pupae of the weaver ant *Oecophylla smaragdina* are added to fish soup and as a supplement they provide extra flavour and texture. A few adult ants, which have a sour flavour, are added as a condiment in similar fashion to using lemon on fish [39].

However, little is known about the nutritional composition and health implications of these different preparations, nor about their frequency of consumption in the societies that eat them. A review of the nutritional content of insects used as food worldwide revealed that while some are a protein-rich food source, others are extremely high in fat [40]. An analysis of the nutritional value of edible insects in different health scenarios suggests that insects have the potential to be conducive to better nutrition, but that some could also be detrimental in certain dietary scenarios due to their high saturated fat content [41]. This, however, is speculative, as there is currently little data available about the place of insects in contemporary diets. Some figures have been published estimating the frequency of traditional insect consumption, however. For example, among the Tukanoan Indians in the northwest Amazon, Dufour [42] found that 12% (for men) and 26% (for women) of animal protein in the diet was provided by edible insects.

The extent to which insects are consumed in any given part of the world is largely constrained by availability, which in turn is increasingly constrained by human influence. Heavy pesticide use is causing a continuing decline in insect populations worldwide, with 67% of invertebrate populations showing a 45% mean decline [43]. However, pesticide use is not ubiquitous, and some species are still highly abundant when in season, and can thus be eaten in large quantities (e.g., various caterpillars in sub-Saharan Africa; [44]), while others are a rare treat (e.g., *Vespula* wasps in Japan; [45]). The overwhelming majority of edible insects are highly seasonal, and in many parts of the world at least one species of edible insect is available at any one time in the year (see e.g., Table 2.2 in [44]).

Yet, year-round consumption of a single species is also possible: For many insects found in large quantities, preservation techniques such as smoking and drying are used to conserve them beyond their season and there is ample potential for improving these techniques for longer preservation [44]. Use of insects as food is also complementary to the use of more well-known food sources. For example, when fish and game availability is low, insect consumption is high, and, when fish and game availability is high, insect consumption is low [42]. Insects may thus be consumed out of need, opportunism, and personal preference, and these reasons vary with the season and by species.

Importantly, edible insects also provide indirect contributions that meet human nutritional needs. Honey is major example of this, and the calories supplied by honey bees are widely recognised to be a significant source of nutrition, particularly among forager groups [46]. In Southern Africa, the kaolin-rich soil generated by termite mound construction is a major source of edible clay, which is particularly important for pregnant women in this region [47].

### 2.2. Income

Insects regularly fetch high prices when sold at markets, higher than the crops from which they were collected and often higher than conventional fish and meat (e.g., chicken, pork, and beef) [48,49]. Weaver ant larvae and pupae sell for about US$12 per kg in Laos and their sale can account for up to 30% of annual household income in rural Thailand [50]. Grasshoppers in Mexico sell for US$13 per kg, and wasp nests sell for US$100 per kg in Japan [51]. Trading in edible insects harvested from agricultural systems can be a lucrative business, sometimes even to the extent that the insect becomes the primary product, while the status of the plant crop is reduced to that of a feed crop or by-product. This is the case for some farmers in Thailand with Patanga grasshoppers (*Patanga succincta*) that feed on maize [36], and similarly for some farmers in Mexico who choose to grow alfalfa in order to harvest edible grasshoppers (*Sphenarium purpurascens*) from their fields [52].

Conventional silkworm farming (with *Bombyx mori*), which requires large areas of land to be set aside for mulberry trees, is a notable example in which the insects have remained a by-product of the system for millennia. Yet the additional income provided by these edible insect is far from insignificant. The trade was worth about US$50.8 million in 2004, generated among about 137,000 households [53]. Such figures have triggered a commercial interest in Madagascar where edible wild silkworm pupae are now being promoted as additional income to silk production [54]. Edible insects collected from agricultural systems thus often serve as a livelihood diversification strategy providing multiple income-generating opportunities [44], which even have the potential to exceed the profits generated by the crops themselves. Thus the collection of edible insects from agricultural systems can enable farmers to develop a multi-production system, which is known to be a more resilient strategy for the smallholder farmers who produce the majority of the world’s food [55].

## 3. Regulating Services

Regulating services are those that regulate the surrounding ecological community, thus maintaining an ecosystem that is well-equipped to consistently deliver marketable services [9]. Edible insects in agricultural systems do this through pollination, and through the control of crop pests.

### 3.1. Pollination

Global agriculture relies to a great extent on insects for pollination and this pollination service is of significant economic value [56]. The most significant edible insect pollinators are the Apidae family of bees, which are one of the most geographically widespread edible insect groups [44]. Both the adults and brood (i.e., larvae and pupae) of bees are used as food in Asia, North and South America, Oceania, and recently Europe [57,58,59,60]. Bees and bee brood collected for food may come from the wild and may be kept in hives [58], which have been used by humans for millennia to keep bees. Honey bees (*Apis* spp.) in particular are now found worldwide in high quantities [61]. Honey bees are also known to increase yields of 96% of agricultural crops [62], and bee pollination in the US alone has been valued at US$3.07 bn [28]. Although bee colonies show signs of decline of up to 30% causing alarm worldwide [63], the consumption of bee brood does not necessarily threaten bee numbers. This is because many beekeepers routinely remove a proportion of bee brood in order to protect colonies against the destructive varroa mite (*Varroa destructor*), a sustainable management strategy that can help guard against colony collapse [60].

Butterflies and moths are other important edible pollinators of agricultural crops. The widely distributed sweet potato horn worm *Agrius convolvuli*, a hawk moth, is reported to be an important pollinator of papaya in Kenya [64]. This importance may very well also be the case in Southeast Asia where *A. convolvuli* also occurs and papaya is an important crop [65]. In Africa, the caterpillars of *A. convolvuli* are eaten [66]. They are found on crops including sweet potato, groundnut, taro, morning glory, lima bean, cowpea, and sunflower, where they may become a pest problem [65]. In Asia, adults may be eaten fried. However, since hawk moths are excellent flyers and difficult to catch, hawk moth consumption is rare. Although edible butterflies and moths are not being actively managed in agricultural production systems, the adults do provide important pollination services while the caterpillars may be a pest. The outcome of this interplay between food, pest, and pollination has not been investigated for edible Lepidoptera.

### 3.2. Biological Control and Animal Community Regulation

Biological control of pests by insects provides significant economic and environmental benefits [28], and many edible insects consume insect pests. A key example of this is the weaver ant (*Oecophylla* spp.), which is a highly abundant and territorially dominant generalist predator that plays an important role in animal community regulation and pest control in a variety of valuable tree crops including mango, citrus, and cashew [67]. Weaver ants are found in Southeast Asia and northern Australia (*O. smaragdina*) and sub-Saharan Africa (*O. longinoda*) [67]. Though rarely or not eaten in Africa [68], the Asian weaver ant (*O. smaragdina*) is one of the most common insect foods in Thailand and Laos [36]. The large-sized larvae and pupae, destined to become new queens, are favoured while the sour tasting adult ants are used as a condiment [39]. Offenberg [69] advocates the use of weaver ants as food and as pest control. For example, both in Thai pomelo and Vietnamese mixed citrus (pomelo and orange) the presence of weaver ants increased crop yields in comparison to absence of ants. Yields were, however, equal between ant control and chemical control of pest insects; yet this is not always the case and the use of chemicals do often give higher yields than the use of weaver ants. However, because ants are a much cheaper control method than chemicals, the end result is a profit gain of up to 47% for the Thai and Vietnamese farmers [69]. This does not include profit gains from trade in weaver ants for food. Key issues herein are that (1) using the larvae and pupae as human food does not impede the biological control capacity of the adult workers that are harvested in very minor quantities and (2) the egg-laying queen remains untouched [39]. Many plantation owners welcome the establishment of colonies in their crop trees and benefit from this multi-production system, notably in economic terms.

Edible wasps are also important predators of crop pests. The edible wasp *Vespula* spp.—which is found in or near to mixed grain and vegetable farming systems throughout Asia, Oceania, North America, and Europe—is a generalist predator and consumes many common crop pests [70]. The wasp larvae are harvested as food after the crop harvest, thus not affecting their efficacy as pest control agents. Estimates of the economic value of wasps to agriculture are unknown, but likely to be high given the quantities of insects they consume: *Vespula* spp. have been known to consume 1.4–8.1 kg/ha/year of prey in temperate climates, and 26.6–99.0 kg/ha/year where hibernation is not practiced [70]. In the US, where wasps are a major predator of crop pests, Losey and Vaughan [28] estimate the control of pests by carnivorous insects at US$13.6 bn. Unfortunately, farmers in Japan are finding that wasp nests, which were once common on the edges of rice fields, are now located increasingly far into the forests, a change that is believed to be a consequence of widespread pesticide use [45].

## 4. Supporting Services

Supporting services are those that ensure other ecosystem services can function [9]. Edible insects in agricultural systems enhance water infiltration, encourage soil formation, and maintain the nutrient cycle. These interconnected processes support the primary productivity of agricultural crops. Several studies have shown that the removal of insects such as ants and termites results in an overall yield decrease of 27%–50% [71,72].

### 4.1. Water Infiltration and Water Retention

Leafcutter ants (*Atta* spp.) are a delicacy in several countries in South America; termites (*Macrotermes* spp.) are consumed as food throughout Southern Africa and Southeast Asia [68]. These insects are found within agricultural landscapes, and in the case of termites, smallholder farmers in many countries welcome the presence of termite mounds in their fields. Soil dwelling ants and termites enhance water infiltration. In arid tropical agricultural systems, water infiltration and retention is particularly problematic. A controlled experiment has suggested that due to improved water infiltration, the presence of ants and termites in such climates can significantly improve crop yields [73].

### 4.2. Soil Formation and Nutrient Cycling

Healthy soils that contain mineral elements needed to maintain life are the essential for regulating all other ecosystem services [74]; therefore the formation of soil and cycling of nutrients back into the soil are crucial regulating services that some edible insects perform.

Termites and ants are important soil engineers. They are surface foragers yet they dwell in the soil (or in the case of some ants, arboreally), and therefore they transport large quantities of nutrient-rich vegetable and animal matter from above ground. Their actions create accumulations of organic matter, which increase and concentrate soil nutrients that are important for maintaining soil fertility. While ants do this by spreading naturally occurring patches of unevenly fertile soil, termites create new fertile soil by decomposing organic matter. Therefore, this is another way in which the presence of termite and ant communities in agricultural landscapes can support and enhance agricultural productivity [73,75]. This is recognized by farmers in parts of Africa and Asia who regularly harvest parts of termite mounds in order to spread the soil across their fields as fertilizer.

In addition to termites and ants, several other edible insect species living in agricultural systems are herbivorous and live above ground. These include Orthopteran and Lepidopteran insects such as grasshoppers (*Oxya* spp., *Sphenarium purpurascens*), locusts (*Locusta migratoria*), and shea caterpillars (*Cirina* spp.), all of which occur in large quantities in certain agricultural landscapes, as indicated in Table 1. The caterpillars consume the leaf matter of trees in agroforestry systems, while Orthopteran herbivores consume both crops and weeds in agricultural fields. Grasshopper and locust presence in fields is known to negatively affect yield [13,76]. The shea caterpillar is also thought to have a detrimental impact on shea nut productivity, though this relationship is yet to be confirmed [77]. However, in non-agricultural tropical ecosystems, invertebrate herbivores are known to assist in nutrient cycling, and liberating nitrogen and phosphorus from tree species [78]. Since both of these minerals are common fertiliser ingredients, tropical herbivory by edible locusts, grasshoppers and caterpillars in agricultural systems may act, through nutrient cycling, as a natural fertiliser. The benefits of this are likely to be minor relative to crop losses incurred by Orthopteran pests, but in agroforestry systems, herbivory, through the addition of fecal matter to the soil, may contribute to soil fertility.

Another important example is the palm weevil, which is found in palm plantations and used as food in South America (*Rhynchophorus palmaraum*, *Rhinostomus barbirostris*), Africa (*Rhynchophorus phoenicis*, *Rhynchophorus bilineatus*), and Southeast Asia (*Rhynchophorus ferrugineus*) [79]. Fallen trees and unworked portions of trees cut to harvest starch are used by weevils that deposit their eggs either directly on the inner tissue or on the trunk. The larvae burrow through and feed on the inner tissue thus accelerating decomposition and aiding nutrient cycling and soil formation [15].

### 4.3. Primary and Secondary Production

In the course of their life cycles, all edible insects accumulate and concentrate energy and nutrients. Some individuals die and decompose, feeding these nutrients back into the soil in a more concentrated form. Others are consumed by insectivorous animals, including humans. All insects therefore contribute to primary and secondary production. Though this is something they share with all other organisms, insects are particularly notable due to their high food-biomass conversion ratio. When insects consume and digest animal or vegetable matter, they store a significantly higher proportion of the energy provided by their food compared to mammals. For example, controlled experiments have suggested that the food conversion ratio is 12-fold more efficient for crickets than for cattle [30]. There are two important reasons for this. The first is that mammals—nearly all of which are homeothermic—must use a large amount of energy to maintain a stable internal body temperature, while poikilothermic insects have an internal temperature that varies with external influence and does not require energy to maintain. The second reason is that insects have extremely fast and short life cycles, and high rates of reproduction per individual, compared to longer-lived mammals, which invest a far greater proportion of energy in reproduction. Insects therefore generate biomass at a far higher rate. Therefore, the accumulation and concentration of energy by edible insects is a particularly significant contribution to both primary and secondary production.

## 5. Cultural Services

Cultural ecosystem services are those that provide ‘recreational, aesthetic, and spiritual benefits’ [9]. Edible insects in agricultural systems are a rich source of such benefits, notably in their contributions to cultural identity, artistic endeavour, folklore and education.

### 5.1. Cultural Identity

In many parts of the world, edible insects are celebrated as an integral part of local identity. An important example of this is the edible grasshopper *Sphenarium purpurascens.* These grasshoppers are eaten in many states in Mexico, but the state of Oaxaca has claimed them as emblematic of Oaxacan culture. At tourist sites in Oaxaca, souvenirs depicting the grasshopper are sold alongside the insects themselves. In other parts of the world, events celebrating edible insects highlight their importance as part of regional cultural identity. For example, in Burkina Faso, the Bobo region is known for its shea caterpillars, and an annual shea caterpillar festival highlights the importance of this edible insect to the communities that eat it. The same is observed in Japan, where several towns and villages throughout the central region hold annual wasp festivals. Significantly, these festivals often use very localized colloquial terms for the wasps themselves, highlighting their importance for community identity formation even on the village level [45].

### 5.2. Art and Folklore

**Figure 2:**
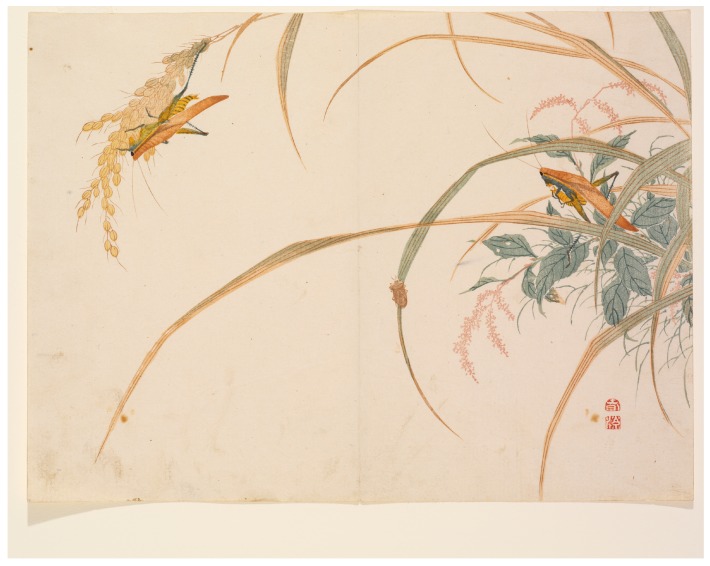
Grasshopper and bee, Things creeping under hand. Woodblock print by Mori Shunkei, 1820. [80].

Celebration of edible insects has also found expression in art and folklore worldwide. Two examples of this that involve edible insects found in agricultural systems include the appearance of the rice grasshopper (*Oxya* spp.) in Asian art and poetry, and the role of the termite in African folklore. Asian art often expresses seasonality, and as such the seasonally available grasshopper, which is traditionally collected from agricultural fields in September, is a recognized symbol of the end of summer and beginning of autumn (Figure 2).

The poet Kobayashi Issa in several of his well-known haiku—a form of poetry that always includes a seasonal reference—also uses the grasshopper to indicate this time of year:

*A cool breeze*
*The grasshopper singing*
*With all his might*
*Good friend grasshopper*
*Will you play*
*The caretaker*
*For my little grave?*
*Giddy grasshopper*
*Take care…Do not*
*Leap and crush*
*These pearls of dewdrop*

The grasshopper is a positive, playful presence in Issa’s poetry. Similarly, termites play a positive and helpful role in African folklore. In parts of West Africa, the presence of a mound can prompt the recounting of traditional tales [81]. One such tale is the Dogon origin myth, which is the story of a God with two wives, one of whom is a termite. The termite controls the flow of water in the creation of the world, and is named ‘the water drawer of God’, in an echo of the known function of termites in influencing water infiltration. Termite mounds are also given spiritual significance in parts of Kenya and Tanzania, where the mounds represent a transformative spirit world, or sexuality and the power of procreation [22].

### 5.3. Education and Recreation

The practice of collecting edible insects is often done by women and children, and in the process, young children are given an insight into the nature of the ecosystems in which they live. For the majority of cultures in which this occurs, such education is not explicit. However, in Japan where collectors do not rely on edible insects for their income, the educational benefits of insect collection are more formally recognized, albeit on a small scale due partly to diminishing numbers of available insects [51]. Until the late 1980s, it was common for children in rural schools to be taken by their teachers to collect grasshoppers in neighboring fields as a class activity on a late summer day. Similarly, in parts of central Japan, educational wasp hunting trips are offered as family activities for both local children and visiting urban tourists. For adults in Japan, wasp hunting is now often practiced as an enjoyable hobby, comparable to fishing or hunting that enhances practitioners’ understanding of, and connection to, the natural environment [51]. Recently, as edible insects have received increased attention in mainstream media, many science outreach events have educated the public about edible insects and their place in ecosystems worldwide. Examples include, but are by no means limited to, a series of Wellcome Trust-funded events run as part of London’s Pestival in 2013 [82], wine tasting with edible insects at the Natural History Museum in London in 2015 [83], and bug banquets held annually at Montana State University since 1988 [84].

### 5.4. Edible Insects as Educators

We can, and do, learn a great deal from the behaviour of edible insects. One example of this is their role as bioindicators of environmental change, which facilitates appropriate management of other ecosystem services. Their high sensitivity to biochemical change means that insects can alert human communities to atmospheric and climatic variation in its early stages [85]. For example, the collection and consumption of aquatic larvae in central Japan has informed awareness of eutrophication and pollution in nearby lakes [86]. Another example of insects as educators is the major role played by social insects in informing efficient design in architecture [87] and engineering [88]. In Zimbabwe, the ventilation system in mounds built by Macrotermes colonies has been mimicked in the design of buildings in Harare, Melbourne, and London, which consequently use less than 10% of the energy of similarly-sized conventional buildings [89]. Similarly, an algorithm developed from observing weaver ants known as Ant Colony Optimisation has been used for diverse purposes including the design of efficient waste [90] and irrigation [91] systems. It is perhaps no coincidence that the insect species occurring in large enough quantities to be exploited as food are often successful social and ecological engineers.

## 6. Ecosystem Disservices

The harmful or costly impacts of ecosystems on humans are often referred to as ‘ecosystem disservices’, and they are crucial to understanding the overall impacts of ecosystem services [92]. Edible insects in agricultural systems are capable of multiple disservices, including crop consumption, threatening human health, and spreading disease. Crop consumption in particular may be so severe that it entirely negates any ecosystem services provided by edible insects. However, to our knowledge there is not a single study that quantifies both the services and disservices contributed by edible insects, and without this it is impossible to know whether or not their presence has a net cost or benefit, nor the extent to which using insects as food may reduce the costs of their disservices to agriculture.

### 6.1. Crop Consumption Leading to Yield Loss

The majority of edible insects in agricultural systems are crop pests, and are there precisely because agriculture creates large areas of concentrated food sources. Of the known edible insects, Orthopteran pests are the most widespread and most destructive. The edible grasshopper *Sphenarium purpurascens* is one of the most important crop pests in Mexico and consumes a wide range of crops [10]. The desert locust *Schistocerca gregaria* is estimated to destroy crops to the value of US$2.5 bn and hundreds of thousands of tons of grain [93]. It seems likely that the extent of these yield losses and the devastating effect they can have on farmers’ livelihoods far outweigh any supporting services contributed by Orthopteran pests. However, the income that harvested Orthopteran pests represent is unknown, and likely to change with fluctuations in demand for edible insects. The time and equipment cost of harvesting and processing these insects may also reduce their value in providing provisioning services. Overall, although it has been suggested that harvesting crop pests could negate their costs to agriculture [93], this has never been satisfactorily quantified within a single agricultural system.

The Lepidopteran shea caterpillar (*Cirina butyrospermi*) is another example of a herbivorous edible insect, but it feeds only on the leaves of the shea tree. Shea trees are common throughout West African agroforestry systems, and shea caterpillars can defoliate entire trees during their short period of abundance in July/August. One study suggested that shea nut production was significantly lower for trees that had been defoliated in the preceding year [94]. Yet this does not necessarily mean they accrue a net cost. There are farmers in West Africa who claim to have a higher income from selling caterpillars – which also require less processing—than selling shea products, suggesting that in some areas the caterpillar may in fact be the preferred crop [95].

### 6.2. Harm to Humans

The majority of edible insects in agricultural systems are not directly harmful to humans. However, a notable exception to this are Hymenoptera such as ants, hornets, wasps, and bees, which are capable of inflicting pain and can—in rare cases of a venom allergy—result in death. Hornets, wasps, and bees do this through venom injection, while weaver ants bite the skin and spray acid into the wound. There is a scale used to measure Hymenopteran stings that ranges from “no pain” to “traumatically painful” [96]. Although allergic reactions are rare, affecting only 2.2% [97] to 3% [98] of studied populations, incidence of death from Hymenoptera stings is high compared to deaths caused by other wild animals.

### 6.3. Disease Vectors

Insects may be vectors of diseases. Among the edible insects discussed in this article, the only disease vector of which we are aware is the palm weevil, *Rhynchophorus* spp., which is a vector of the destructive red ring disease (RRD), *Bursaphelenchus cocophilus*. RRD causes palms to yellow and eventually die, or to produce stunted leaves, and it is major threat to yields in oil and coconut palm plantations. The palm weevil is its only known vector, and targeting the weevil itself is widely considered the only way to combat RRD [99].

## 7. Discussion

This paper has given an overview of some of the ecosystem services and disservices provided by edible insects found in agricultural systems, with examples to illustrate these services.

It is no coincidence that so many of the world’s commercially available insects are those found in existing agricultural systems: The spread of agriculture has also enabled many of the edible insects that accompany it to occur at higher densities than in wild landscapes. Perhaps it is thus also no coincidence that humans worldwide find these insects so palatable. Many of them do, after all, consume parts of the food plants that we have cultivated for our own consumption over millennia. Agricultural fields are thus excellent edible insect resources, as the insects are available in aggregations, and can be collected in large quantities with high energy-efficiency and low risk accrued to harvesters. Perhaps our relationship with our crops and the edible insects that live among them could best be described as symbiosis, a mutualistic interaction with an ancient history.

Yet current management practices are focused on the destruction of most of insects that co-occur with agriculture. Integrated Pest Management (IPM) strategies are a notable exception to this, and within IPM the maintenance of edible insects in agriculture has been advocated as a possible novel management direction [100]. For this to be realized, a more in-depth understanding and quantification of the ecosystem services and disservices obtainable from edible insects will certainly be necessary. For example, in Mexico an experimental study found that alfalfa plots where grasshoppers were collected for food had fewer oothecae (egg cases) when compared with control plots, presumably leading to less severe consequent outbreaks [13]. Plots where insecticide was used had even lower oothecae densities. While this study shows the potential efficacy of insect collection for pest control, it lacks systematically collected data on yield or income that would allow a comparison between the financial returns from grasshoppers with the loss of alfalfa yield, vs. the costs of pesticide use [52]. This is crucial, since even trials of pesticides used against non-edible insects have found mixed results in terms of the overall costs and benefits of pesticide use [101]. A later study modeled the potential biomass available from the harvest of Mexican grasshoppers, if pesticides were abandoned [93]. This study found that 350,000 tonnes of grasshoppers could be obtained, potentially supplying nine million people with a year’s supply of necessary dietary protein and US$350,000 of income, as well as reducing health problems from pesticide use. However, no estimates were offered with regard to yield losses nor labour costs to these farmers, because the yield losses and labour costs incurred by a strategy of grasshopper collection instead of pesticide use have not been quantified. Furthermore, the longer term effects of grasshopper collection have not been monitored, and the sustainability of the grasshopper harvest over time could depend heavily on harvesting intensity. Finally, the impact of increased grasshopper supply and reduced alfalfa supply on market prices for these commodities could be significant, and could influence whether or not farmers benefit from harvesting these insects. Overall, it is likely that we could learn a great deal from combining such knowledge with traditional management strategies that support the edible insect populations while also boosting crop production.

Weaver ant (*Oecophylla* spp.) biological control and use as human food is an important example of this. Research suggests that harvesting the larvae and pupae, while very few worker ants are removed, causes the worker ants to increase production of new workers [102]. This response would benefit the colony and its role in the plantation. After all, queen-destined larvae and pupae are those favoured as food, and new adult queens do not contribute to colony survival but leave the colony to establish a new colony [102]. Weaver ant management involves, amongst others, feeding sugar water, not harvesting the queen, connecting trees with strings for easier access by worker ants, and creating sticky barriers on tree trunks to avoid attacks by ground nesting antagonistic ants [69]. Further improvements can be made. The above strategies increase the fitness of colonies within plantations, but not the fitness of those outside the plantation. In addition, colonies do not live forever—surrounding vegetation must be preserved so incipient colonies may be established that can come to inhabit the plantation [24]. Furthermore, because the reproductive females are intensively harvested, colony reproduction of plantation colonies is expected to be low or non-existent. Breeding and raising new colonies indoors is one avenue of research to address this [103].

Another example is that of *Vespula* spp. wasps, which have also been proposed as a candidate for developing novel biological control strategies [70]. Perhaps such strategies could be developed in combination with knowledge of wasp rearing developed by farmers in rural Japan, who keep nests near to their homes and vegetable gardens, in some cases even harbouring thousands of hibernating wasps over the cold winter months in the hope of promoting their survival and increasing wasp numbers [45].

An understanding of the ecosystem services and disservices contributed by edible insects is crucial for developing such strategies. One illustration of this is the case of the edible hawk moth, *A. convolvuli*. The moth is a pollinator of papaya, an important ecosystem service, and forest conservation is required to secure this service [64]. Yet the caterpillars are sometimes a pest of crops, an ecosystem disservice. Incorporating trade in hawk moths as food may improve incentives for conservation [48]. Can management be developed to reap benefits of this triangle? Can hawk moth caterpillars be grown on one crop and the adults used to pollinate papaya and used as food source?

Similarly, palm weevils, found throughout the tropics, provide valuable ecosystem services that may counteract their role as pests. Indigenous peoples in Venezuela, for example, are highly knowledgeable of weevil biology and, given their preference for *R. palmarum* as food, exercise controlled supply of larvae [15]. The *R. palmarum* adults are attracted to exposed inner palm tissue, while *R. barbirostris* adults oviposit on the intact surface of the trunk. The former also arrive more quickly than the latter. By intentionally felling trees and making deep cuts in the trunk, thereby exposing more inner tissue, a higher number of *R. palmarum* grubs can be harvested [15]. Weevils are also reported to oviposit eggs on standing palm trees in which case they are considered a pest, as described above with red ring disease [44]. Choo et al. [17] suggest building on the knowledge and practice of indigenous peoples to aid in weevil control in palm plantations. Perhaps a management system could be developed to attract the weevils to intentionally felled trunks of lower-quality palms, avoiding the infestation of the standing higher-quality palms [104]. The resulting grubs provide additional food and income, as do all the insects reviewed here.

## 8. Conclusions

Although the current focus on advancing the use of edible insects as food lies on production in enclosed systems [44,105], this review highlights the ecological and economic importance of edible insects in existing agricultural systems. This is important because current land management tends to promote the continued expansion of agriculture that relies on agrochemical use and prioritises monocultures [106], thus endangering the existence of these valuable insect species [13,32,51,63,100,107]. There is considerable evidence to suggest that agricultural intensification can increase crop plant yields, thus enabling food production to meet growing demand while also freeing a greater area of land to be devoted to wild nature, decelerating environmental degradation [108]. However, demand for animal protein is a significant element of increasing food demand. If increases in yields of grain used for animal feed come at the expense of destroying protein-rich edible insects that contribute multiple ecosystem services to humans, which is the more economically and environmentally viable strategy?

The answer to this is likely to differ significantly for different systems, particularly given the diversity of crops and insect species that coexist worldwide. We certainly do not argue that all or even any edible insects offer a known net benefit to agriculture via ecosystem services. However, we have discussed several edible insect species that contribute important ecosystem services, suggesting that there may be significant environmental, nutritional, and economic incentives to maintaining edible insects within certain agricultural systems. For example, collecting pest insects as food could reduce both their efficacy as pests and the costs of controlling them. Without clearer comparative data, it is impossible to know whether these benefits outweigh the costs of their ecosystem disservices. Yet another consideration is the changing global climate, which threatens the livelihoods of many of the world’s food producers. Particularly smallholder food producers could benefit significantly from the development of resilient multi-production systems that yield both plant and animal foods. In order to achieve this, we argue that edible insects and the ecosystem services they provide should be considered in the development of agricultural intensification strategies, particularly in tropical settings. Similarly to many research areas in the broad field of insects as food, hard data to guide such programs is currently lacking. To determine the relative costs and benefits of agriculture that incorporates food insects, we recommend conducting comparative life cycle analyses (LCAs) that compare the economic, environmental, and nutritional outputs of grain-livestock systems and crop-insect agriculture. To determine the impacts of adopting crop-insect agriculture on farmers’ livelihoods, we recommend conducting field trials, using a randomized controlled trial (RCT) framework [109], in tropical farming systems. To develop strategies for maximizing benefits and minimizing costs accrued by edible insects, we advocate combining knowledge from traditional management strategies with recent scientific understanding of insect ecology.

Overall, we hope that this review will stimulate a greater interest in the commercial and environmental potential of edible insects in existing agricultural systems. Recent commercial and research interest in insects as food has rarely appreciated the exceptional opportunities that are offered by these insects. We look forward to future research that will elucidate and quantify the costs and benefits accrued by the presence of edible insects in agricultural systems, and to the development of innovative agricultural strategies that will maximize the ecosystem services provided by such insects.

## Figures and Tables

**Table 1:** Summary of commercially available edible insect species closely associated with agricultural systems, and the ecosystem services and disservices they provide.

Species (Colloquial Name, *Scientific Name)*	Region(s)/Countries	Ecosystem Services	Details	Ecosystem Disservices	Farming System(s)	Key Reference(s)
Chapulines, *Sphenarium purpurascens*	Mexico	Provisioning, Cultural, Supporting	Source of income and nutrition; part of regional identity; herbivory assists nutrient cycle	Herbivory with influence on yield	Smallholder grain crops (primarily maize, alfalfa)	[13]
Agave worms, *Comadia redtenbacheri* (red) *Aegiale hesperiaris* (white)	Mexico	Provisioning, Supporting	Source of income and nutrition; aids decomposition	Herbivory with influence on yield	Agave plantations (primarily for pulque and mescal production)	[14]
Palm weevil larvae (*Rhynchophorus* spp.)	Papua New Guinea, Asia, Central Africa, West Africa, South America	Provisioning, Supporting	Source of income and nutrition; aids decomposition	Disease vector (*Bursaphelenchus cocophilus.* red ring disease nematode)	Sago palm groves, oil palm plantations, coconut palm plantations, date palm plantations	[15]
Wasp brood (*Vespula* spp.)	Japan, South Korea, China, New Zealand, Papua New Guinea	Provisioning, Regulating, Cultural	Source of income and nutrition; consumes crop pests and regulates forest animal community; source of education and part of regional identity	Can be harmful to humans	Small-scale vegetable gardens	[16]
Locust (*Locusta migratoria)*	Middle East, Central Africa, East Africa	Provisioning, Supporting	Source of income and nutrition; herbivory assists nutrient cycle	Herbivory with influence on yield	Grain crops	[17]
Cricket (*Acheta* spp., *Gryllus* spp.)	Asia	Provisioning, Supporting	Source of income and nutrition; herbivory assists nutrient cycle	Herbivory with possible influence on yield	Small-scale vegetable gardens, rice paddy fields	[18]
Grasshopper (*Oxya* spp.)	Asia–China, South Korea, Japan	Provisioning, Supporting	Source of income and nutrition; herbivory assists nutrient cycle	Herbivory with influence on yield	Rice paddy fields	[19]
Dragonfly larvae (species unknown), water beetles (*Cybister* spp. and *Hydrophilus* spp.) and other aquatic taxa	Southeast Asia–Thailand, Laos	Provisioning, Regulating	Source of income and nutrition; regulate the aquatic faunal community through predation		Flooded rice paddy fields	[20,21]
Termite (*Macrotermes* spp.)	Southern Africa, Central Africa, East Africa, Southeast Asia	Provisioning, Supporting	Source of income and nutrition; soil manipulation aids water infiltration and herbivory assists nutrient cycle	Herbivory with possible influence on yield	Mixed smallholder crops, palm plantations	[22]
Shea caterpillar (*Cirina butyrospermi*)	West Africa	Provisioning, Supporting	Source of income and nutrition; herbivory assists nutrient cycle	Herbivory with possible influence on yield	Mixed agroforestry systems (Maize, millet, cotton, etc)	[23]
Weaver ant (*Oecophylla smaragdina*)	Asia	Provisioning, Regulating, Supporting	Source of income and nutrition; consumes crop pests and regulates herbivory, fruit damage and pollination; herbivory assists nutrient cycle	Negative effect on host tree productivity and pollinator abundance	Tropical plantations (e.g., mango, citrus, cashew)	[24]
Leafcutter ant (*Atta* spp.)	South America	Provisioning, Supporting	Source of income and nutrition; herbivory assists nutrient cycle	Herbivory with influence on yield	Tropical tree plantations (e.g., citrus, cocoa)	[25]
